# Supplementary figures and images for: Simulating Serial-Target Antibacterial Drug Synergies Using Flux Balance Analysis
Source: PLoS One. 2016 Jan 28;11(1):e0147651. doi: 10.1371/journal.pone.0147651 (PMC4731467; doi:10.1371/journal.pone.0147651)

S1 Fig

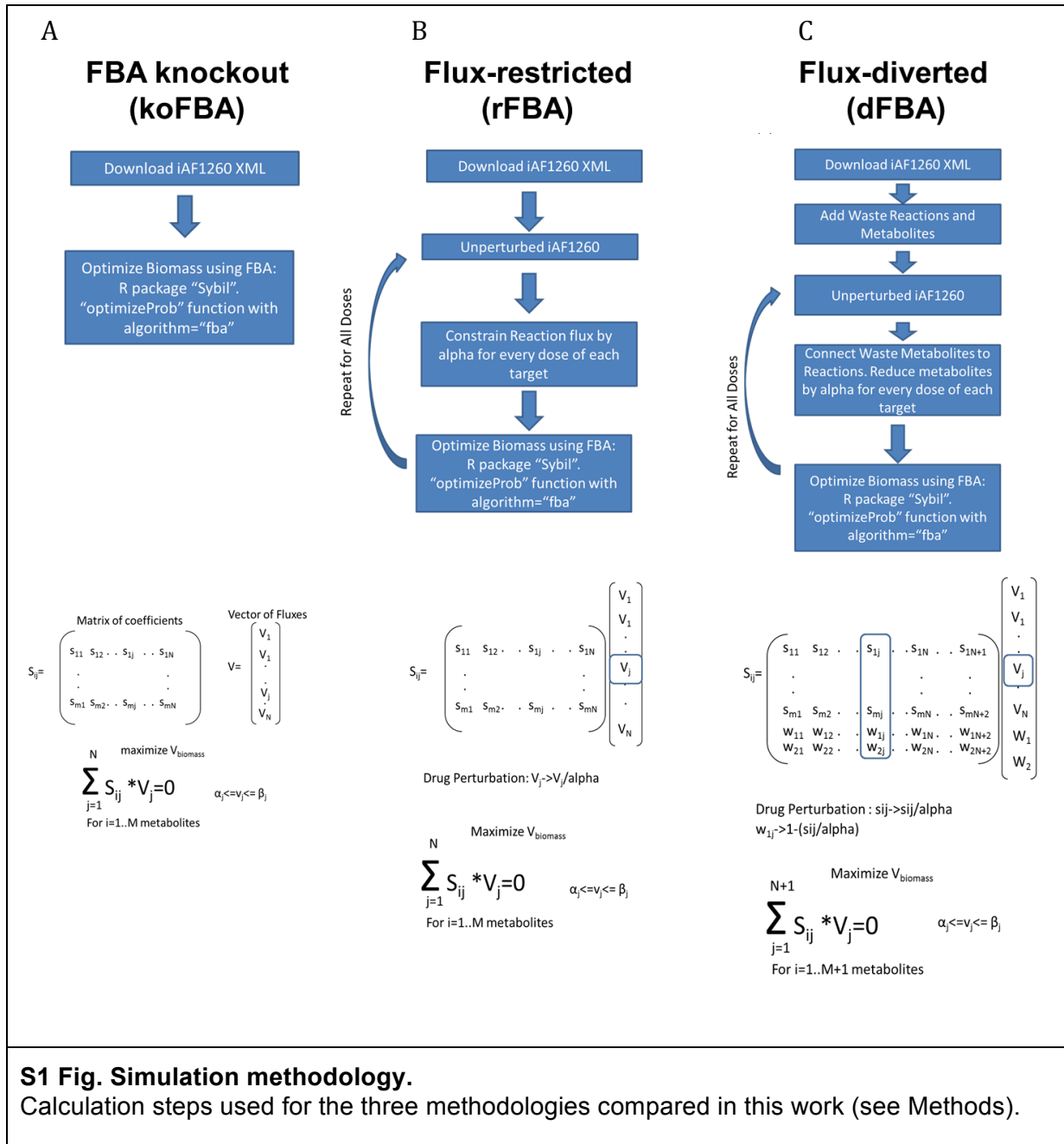

Supplement: S1 Fig — Calculation steps used for the three methodologies compared in this work (see Methods). (PDF) [file pone.0147651.s005.pdf]
