# Supplementary material for: Simulating Serial-Target Antibacterial Drug Synergies Using Flux Balance Analysis
Source: PLoS One. 2016 Jan 28;11(1):e0147651. doi: 10.1371/journal.pone.0147651 (PMC4731467; doi:10.1371/journal.pone.0147651)
Supplement: S2 Fig — Each symbol represents the simulated FBA-ko response to inhibiting a single or pair of targets (see Fig 2), with enzymes organized by pathway and metabolic reaction order in the iAF1260 model. Symbol shape shows the type of interaction (synthetic lethal “SL”, partial synthetic lethal “PSL”, potentiation “Pot”, or non-interaction “None”), size shows the effect max(I), and color shows the synergy max(ΔI) between two targets. The only strong interactions found are SL between “interchangeable” enzymes along alternative paths to a common endpoint, and there are no synergies between two singly essential enzymes. (PDF) [file pone.0147651.s006.pdf]

S2 Fig

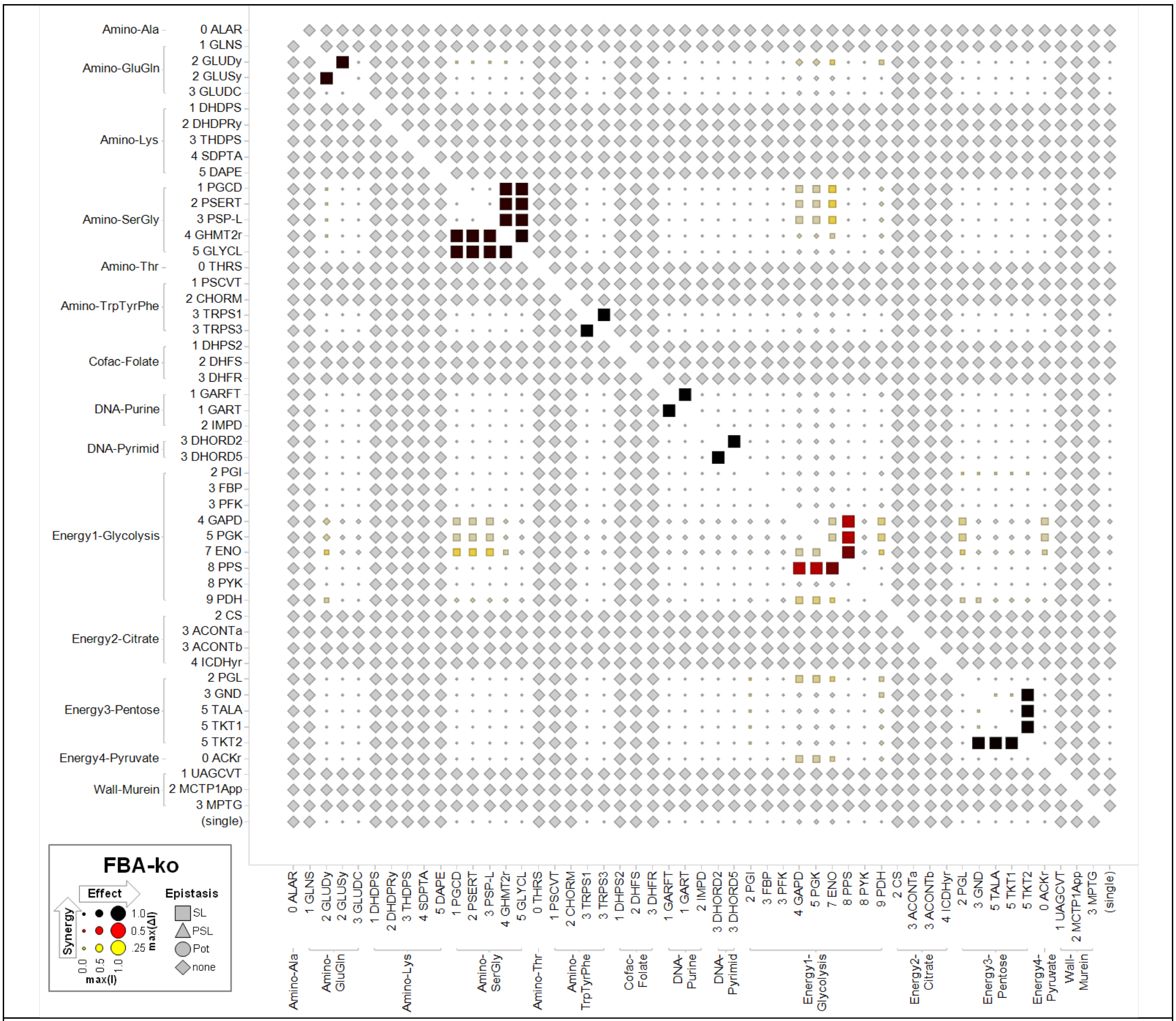

S2 Fig. Mechanistic patterns in FBA-ko simulated inhibitor combinations.
Each symbol represents the simulated FBA-ko response to inhibiting a single or pair of targets (see Figure 2), with enzymes organized by pathway and metabolic reaction order in the iAF1260 model. Symbol shape shows the type of interaction (synthetic lethal “SL”, partial synthetic lethal “PSL”, potentiation “Pot”, or non-interaction “None”), size shows the effect max(I), and color shows the synergy max(ΔI) between two targets. The only strong interactions found are SL between “interchangeable” enzymes along alternative paths to a common endpoint, and there are no synergies between two singly essential enzymes.
