# Supplementary material for: Simulating Serial-Target Antibacterial Drug Synergies Using Flux Balance Analysis
Source: PLoS One. 2016 Jan 28;11(1):e0147651. doi: 10.1371/journal.pone.0147651 (PMC4731467; doi:10.1371/journal.pone.0147651)
Supplement: S3 Fig — Each symbol represents the simulated FBA-res response to inhibiting a single or pair of targets (see Fig 2), with enzymes organized by pathway and metabolic reaction order in the iAF1260 model. Symbol shape shows the type of interaction (synthetic lethal “SL”, partial synthetic lethal “PSL”, potentiation “Pot”, or non-interaction “None”), size shows the effect max(I), and color shows the synergy max(ΔI) between two targets. The triangles for PSL epistasis point toward the inactive agent in the pairing. Single enzyme effects are very similar to those for FBA-ko. Interactions are also similar to FBA-ko, except that there are some PSL involving one singly essential enzyme. (PDF) [file pone.0147651.s007.pdf]

S3 Fig

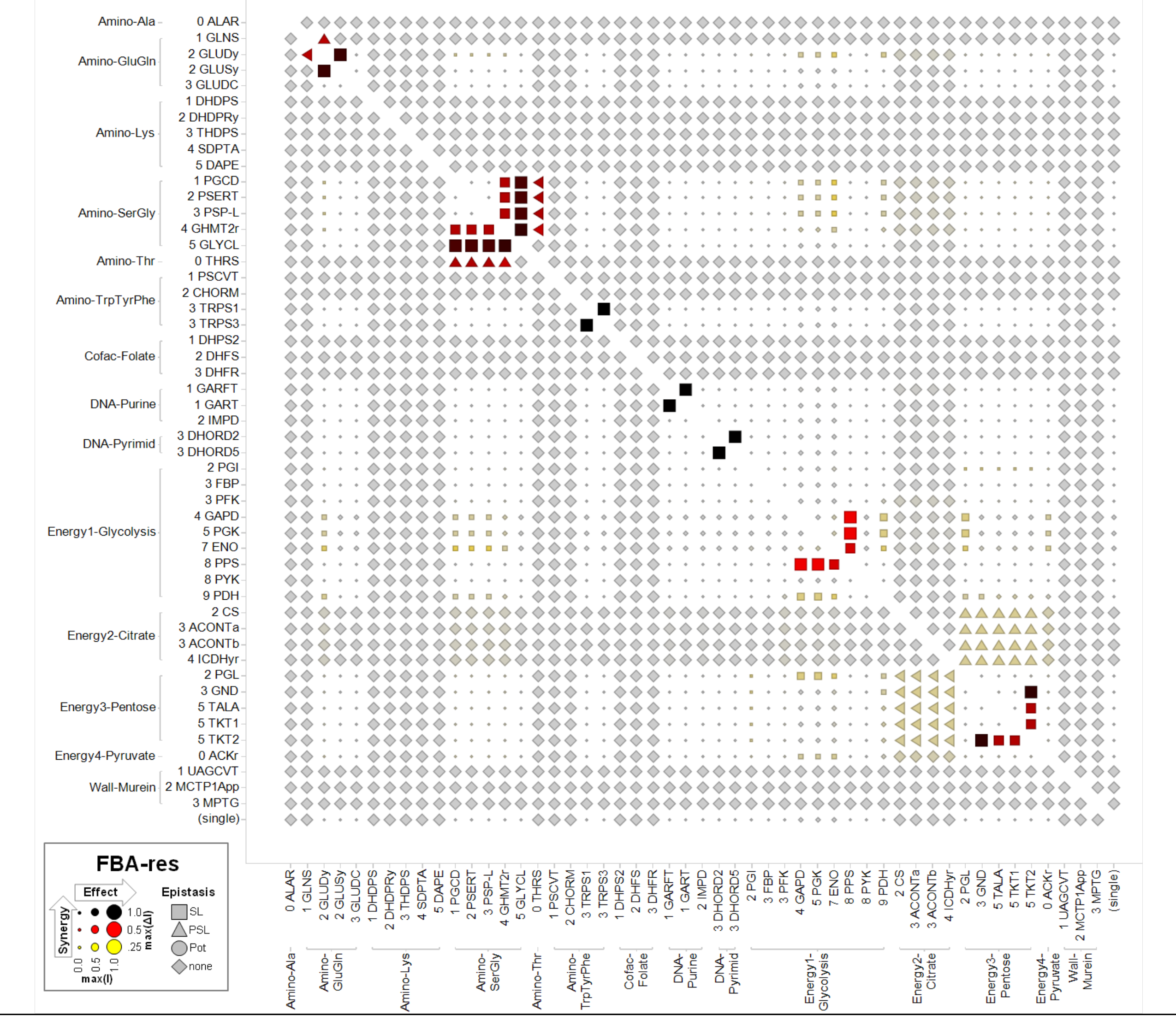

S3 Fig. Mechanistic patterns in FBA-res simulated inhibitor combinations. Each symbol represents the simulated FBA-res response to inhibiting a single or pair of targets (see Figure 2), with enzymes organized by pathway and metabolic reaction order in the iAF1260 model. Symbol shape shows the type of interaction (synthetic lethal “SL”, partial synthetic lethal “PSL”, potentiation “Pot”, or non-interaction “None”), size shows the effect max(I), and color shows the synergy max(ΔI) between two targets. The triangles for PSL epistasis point toward the inactive agent in the pairing. Single enzyme effects are very similar to those for FBA-ko. Interactions are also similar to FBA-ko, except that there are some PSL involving one singly essential enzyme.
