# Supplementary material for: Simulating Serial-Target Antibacterial Drug Synergies Using Flux Balance Analysis
Source: PLoS One. 2016 Jan 28;11(1):e0147651. doi: 10.1371/journal.pone.0147651 (PMC4731467; doi:10.1371/journal.pone.0147651)
Supplement: S4 Fig — Each symbol represents the simulated FBA-div response to inhibiting a single or pair of targets (see Fig 2), with enzymes organized by pathway and metabolic reaction order in the iAF1260 model. Symbol shape shows the type of epistasis (synthetic lethal “SL”, partial synthetic lethal “PSL”, potentiation “Pot”, or non-interaction “None”), size shows the effect max(I), and color shows the synergy max(ΔI) between two targets (same scale as Fig 2). Single agents are shown along the bottom and right edges. The triangles for PSL epistasis point toward the inactive agent in the pairing. Single enzyme effects are very similar to those for FBA-ko and FBA-res, but new patterns emerge for interactions. In particular, the FBA-div simulations yield strong potentiation synergies between serial targets in the same pathway that are not found for FBA-res or FBA-ko. (PDF) [file pone.0147651.s008.pdf]

S4 Fig

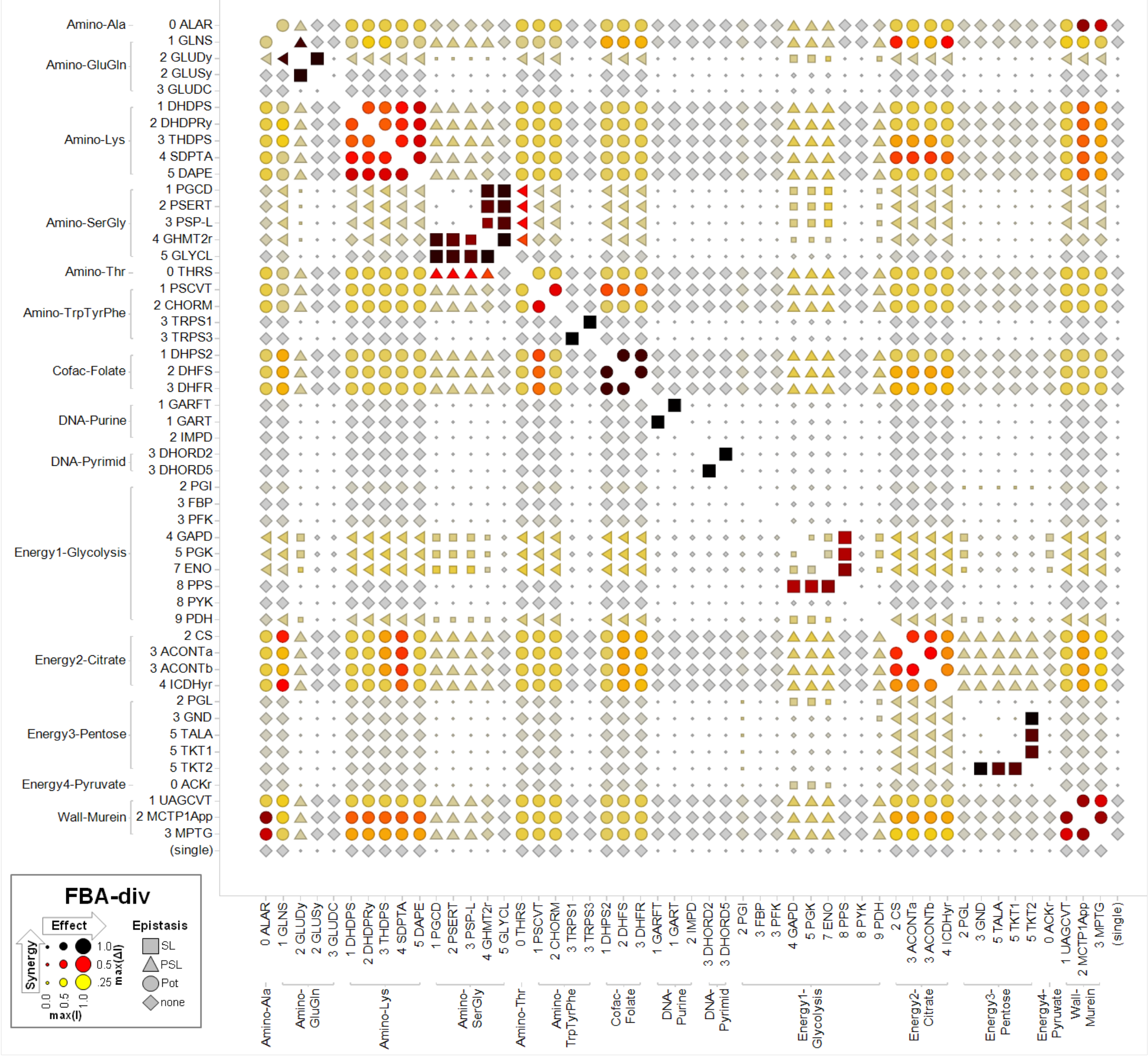

S4 Fig. Mechanistic patterns in FBA-div simulated inhibitor combinations.
Each symbol represents the simulated FBA-div response to inhibiting a single or pair of targets (see Figure 2), with enzymes organized by pathway and metabolic reaction order in the iAF1260 model. Symbol shape shows the type of epistasis (synthetic lethal “SL”, partial synthetic lethal “PSL”, potentiation “Pot”, or non-interaction “None”), size shows the effect max(I), and color shows the synergy max(ΔI) between two targets (same scale as Figure 2). Single agents are shown along the bottom and right edges. The triangles for PSL epistasis point toward the inactive agent in the pairing. Single enzyme effects are very similar to those for FBA-ko and FBA-res, but new patterns emerge for interactions. In particular, the FBA-div simulations yield strong potentiation synergies between serial targets in the same pathway that are not found for FBA-res or FBA-ko.
