# Supplementary material for: Simulating Serial-Target Antibacterial Drug Synergies Using Flux Balance Analysis
Source: PLoS One. 2016 Jan 28;11(1):e0147651. doi: 10.1371/journal.pone.0147651 (PMC4731467; doi:10.1371/journal.pone.0147651)
Supplement: S5 Fig — Experimentally measured drug-drug dose response matrices, ordered by total activity. The effects range from potentiation-like synergies (eg, Sulfx+Trimp), through non-interacting pairs (eg, Nfurz+Trimp), to antagonisms (eg, Ampcl+Sulfx). There were also inactive single agents such as Dfrox and Tcarb for which interactions could not be accurately determined. Synergy scores were calculated and compared to the simulated dose-response matrices. (PDF) [file pone.0147651.s009.pdf]

S5 Fig

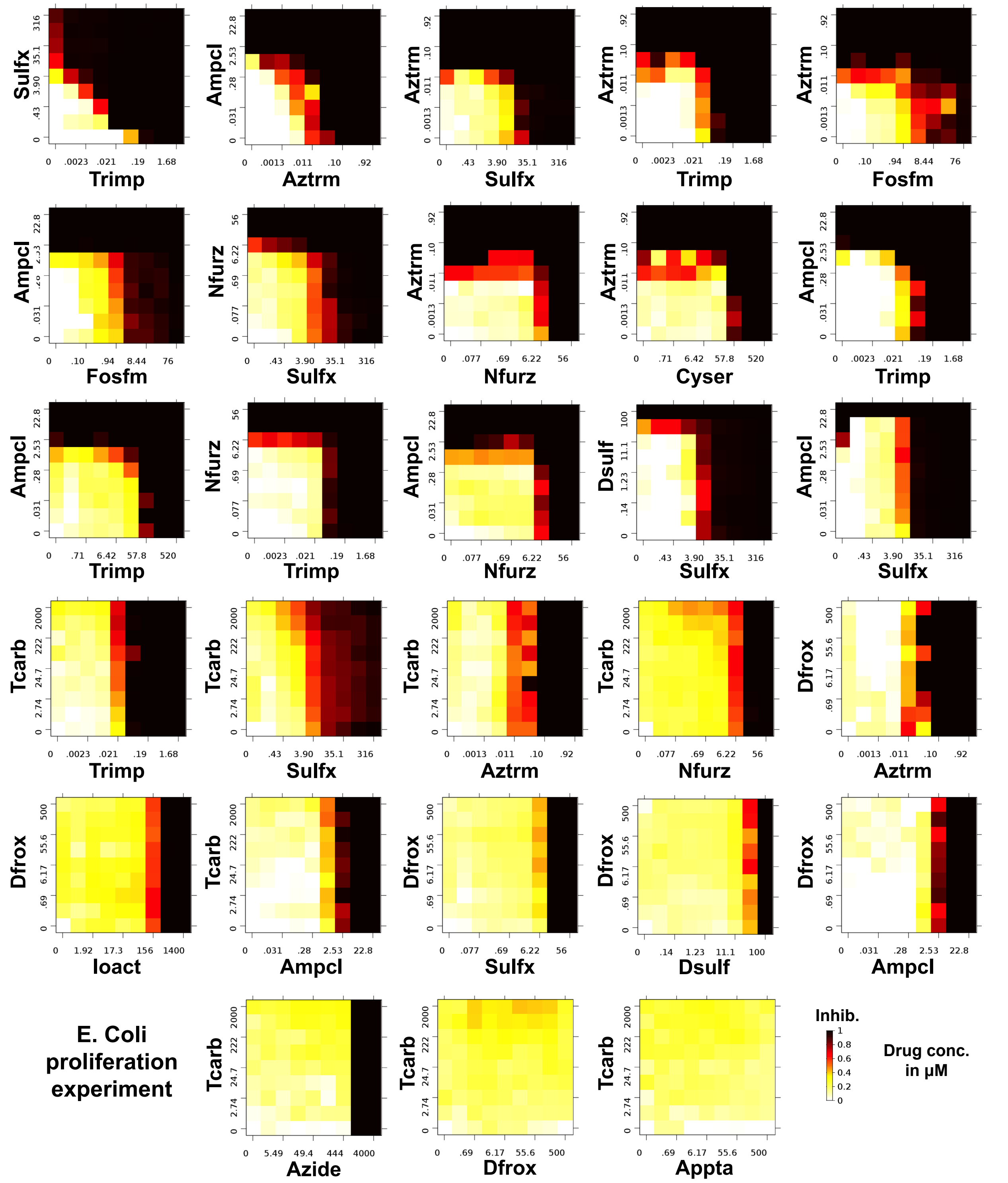

S5 Fig. Experimental dose response matrices.

Experimentally measured drug-drug dose response matrices, ordered by total activity. The effects range from potentiation-like synergies (eg, Sulfx+Trimp), through non-interacting pairs (eg, Nfurz+Trimp), to antagonisms (eg, Ampcl+Sulfx). There were also inactive single agents such as Dfrox and Tcarb for which interactions could not be accurately determined. Synergy scores were calculated and compared to the simulated dose-response matrices.
